# Airway gas temperature within endotracheal tube can be monitored using rapid response thermometer

**DOI:** 10.1038/s41598-021-88787-3

**Published:** 2021-05-05

**Authors:** Shigeharu Nakane, Kennosuke Tsuda, Masahiro Kinoshita, Shin Kato, Sachiko Iwata, Yung-Chieh Lin, Mihoko Mizuno, Shinji Saitoh, Osuke Iwata

**Affiliations:** 1 Department of Paediatrics and Neonatology, Nagoya City University Graduate School of Medical Sciences, Kawasumi 1, Mizuho-cho, Mizuho-ku, Nagoya, Japan; 2 Department of Paediatrics, Daido Hospital, Nagoya, Japan; 3 Department of Paediatrics and Child Health, Kurume University School of Medicine, Fukuoka, Japan; 4 Department of Paediatrics, National Cheng Kung University Hospital, Tainan, Taiwan, ROC

**Keywords:** Respiratory tract diseases, Neonatology

## Abstract

Inappropriate preparation of respiratory gases is associated with serious complications during mechanical ventilation. To develop a temperature monitoring system of respiratory gases within the endotracheal tube, four newborn piglets were studied using an ultra-rapid-response thermometer attached to the closed endotracheal tube suction system. Respiratory gas temperatures were monitored at the mouth-corner level of the endotracheal tube using three thermocouples (T_airway_, inserted into the endotracheal tube via the closed suction system; T_tube_centre_ and T_tube_wall_, embedded within the endotracheal tube 0.5 mm and 1.6 mm from the tube wall, respectively). Univariate analysis showed that inspiratory T_tube_centre_ and inspiratory T_tube_wall_ were positively correlated with inspiratory T_airway_ (both p < 0.001). Multivariate analysis showed the dependence of inspiratory T_airway_ on inspiratory T_tube_centre_ and T_tube_wall_ and deflation of endotracheal tube cuff (p < 0.001, p = 0.001 and p = 0.046, respectively). Inspiratory gas temperature within the endotracheal tube can be monitored using a thermometer attached to the closed endotracheal tube suction system. Our system, with further validation, might help optimise respiratory gas humidification during mechanical ventilation.

## Introduction

For patients who require mechanical ventilation, the inspired gas is heated and humidified to reduce drying of the respiratory mucosa, airway ulceration and impaired secretion clearance^1–3^. Insufficient and excessive humidification of respiratory gases are associated with serious complications, such as endotracheal tube obstruction and ventilator-associated pneumonia (VAP)^1,2,4,5^. The incidence rate of VAP is 0.0–4.4 per 1000 days of mechanical ventilation^6^, which increases the mortality rate by 13% compared to those who do not develop VAP^7^. To reduce such complications, the humidity of respiratory gases is prepared to 44 mgH_2_O/L, or the saturated vapour pressure at the body temperature of 37 °C, within the humidifying chamber^8,9^. Condensation is reduced by heating the respiratory gases to 40 °C before reaching the Y-piece, with the expectation that the gases will cool back down to 37 °C through the non-heated endotracheal tube.

However, the temperature reduction through the endotracheal tube varies according to the ambient temperature and several other settings^10^. Despite the importance of monitoring the humidity of respiratory gases provided to the patient, there is no established method for monitoring the humidity of respiratory gases at the distal end of the endotracheal tube, because non-invasive placement of hygrometers within the endotracheal tube is difficult. In addition, most hygrometers cannot distinguish inspiratory gas humidity from expiratory gas humidity because of their limited time constant.

Although direct monitoring of inspiratory gas humidity appears difficult, the humidity of gases delivered to the patient can be estimated using their lowest temperature before reaching the patient’s airway mucosa. Theoretically, this can be monitored at the distal end of the endotracheal tube at the mouth-corner level, because the gases are deprived of humidity from 44 mgH_2_O/L according to the extent of temperature drop below 37 °C.

We designed the current experimental study to test whether the inspiratory gas temperature can be monitored within the endotracheal tube at the mouth-corner level using an ultra-rapid-response thermometer, which is attached to the closed endotracheal tube suction system, in newborn piglets.

## Results

**Figure 1:**
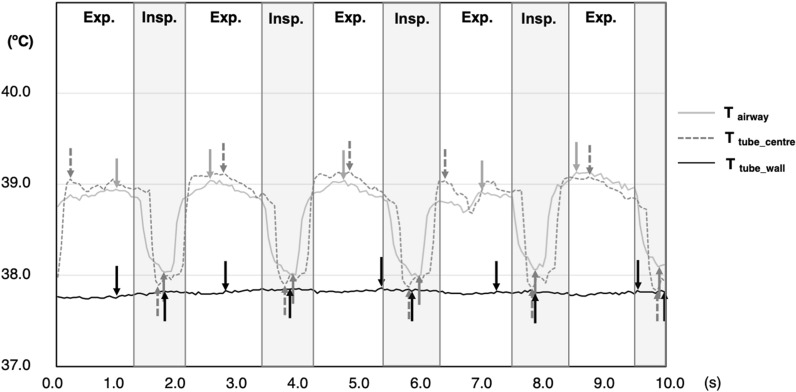
Representative time-trend data of respiratory gas temperatures. A representative waveform recorded with the following ventilator settings: respiratory rate, 30/min; peak airway pressure, 15 cmH_2_O; inspiratory time, 0.5 s; positive end-expiratory pressure, 5 cmH_2_O; and with inflating the endotracheal tube cuff. T_airway_ and T_tube_centre_ showed similar waveforms between each other with distinct temperature changes between inspiration and expiration, whereas T_tube_wall_ showed no apparent temperature changes between inspiration and expiration. *Exp.* expiratory phase; *Insp.* inspiratory phase;  T_airway_ airway temperature; T_tube_centre_ temperature at the centre of the endotracheal tube; and T_tube_wall_ temperature at the wall surface of the endotracheal tube.

Values are presented as the mean (standard deviation) unless otherwise indicated. Twelve temperature data collection cycles were performed from the four piglets (3 cycles per each); one cycle of which was excluded due to a malfunction of the humidifier. When T_tube_centre_ was expressed with time, the highest temperature values were observed at the beginning of the expiratory phase and the lowest during the inspiratory phase (Fig. 1).

### Inspiratory temperature of three different locations

**Figure 2:**
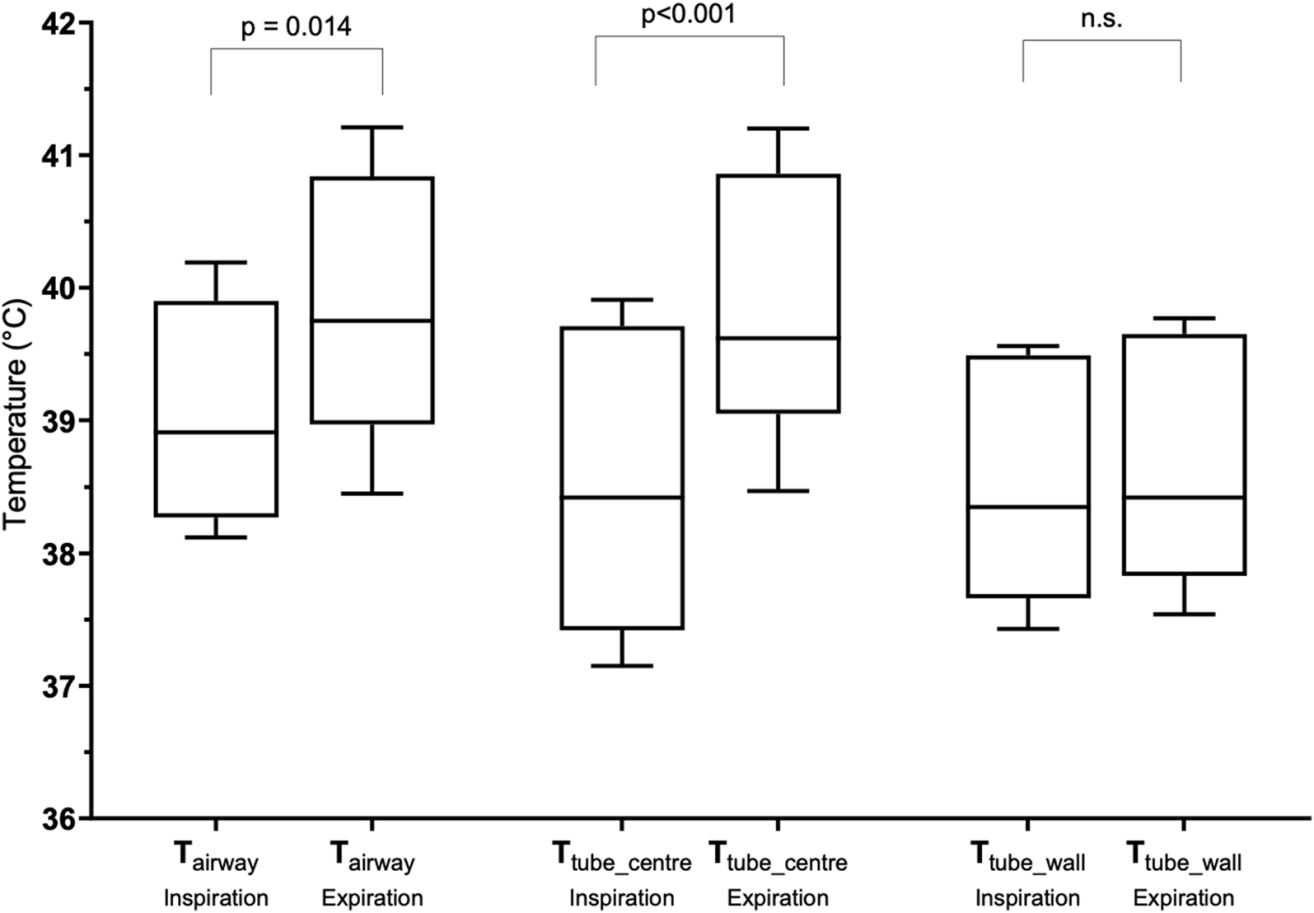
Temperature values recorded at three different locations. T_airway_ and T_tube_centre_ were relatively higher for the expiratory phase than for the inspiratory phase. Symbols: box, first and third quartiles; bold line, median; perpendicular line, range. *T*_*airway*_ airway temperature; *T*_*tube_centre*_ temperature at the centre of the endotracheal tube; and *T*_*tube_wall*_ temperature at the wall surface of the endotracheal tube.

The average inspiratory temperature at the centre of the endotracheal tube (T_tube_centre_), temperature at the wall surface of the endotracheal tube (T_tube_wall_) and airway temperature (T_airway_) were 38.6 (1.0)°C, 38.5 (0.9)°C and 39.4 (1.0)°C, respectively, whereas the average expiratory T_tube_centre_, T_tube_wall_ and T_airway_ were 39.8 (1.0)°C, 38.6 (0.9)°C and 39.8 (0.9)°C, respectively (Fig. 2). (See the “Methods” section for the detail of the temperature measurement).

### Dependence of inspiratory T_airway_ on other temperature measures and ventilator settings

In the univariate analysis, inspiratory T_tube_centre_ and T_tube_wall_ were positively correlated with T_airway_ (both p < 0.001; see Supplementary Fig. S1 online). The peak inspiratory pressures (PIPs) of 15 and 20 cmH_2_O were associated with higher inspiratory T_airway_ (vs. PIP of 10 cmH_2_O, p = 0.013 and p = 0.004, respectively). Inspiratory T_airway_ did not depend on the difference in the inspiratory time and inflation of endotracheal tube cuff. The multivariate analysis showed that higher inspiratory T_airway_ was best explained by higher inspiratory T_tube_centre_, higher inspiratory T_tube_wall_ and deflation of endotracheal tube cuff (p < 0.001, p = 0.001 and p = 0.046, respectively; Table 1).

**Table 1:** Dependence of T_airway_ on other inspiratory gas temperatures and covariates: univariate and multivariate analyses.

Variables	Regression coefficient				
	Mean	95% CI		Wald Chi-square	p
		Lower	Upper		
**Univariate analysis**					
Inspiratory gas temperature					
T_tube_centre_	0.929	0.812	1.045	243.68	< 0.001
T_tube_wall_	1.033	0.921	1.146	324.32	< 0.001
Peak inspiratory pressure					
20 cmH_2_O	1.236	0.404	2.069	8.47	0.004
15 cmH_2_O	0.735	0.157	1.312	6.22	0.013
10 cmH_2_O	1.000			Reference	
Endotracheal tube cuff					
Inflated	-0.523	-1.045	0.000	3.84	0.050
Deflated	1.000			Reference	
Inspiratory time					
1.0 s	0.120	-0.008	0.247	3.38	0.066
0.5 s	1.000			Reference	
**Multivariate analysis**					
Inspiratory gas temperature					
T_tube_centre_	0.643	0.427	0.858	34.17	< 0.001
T_tube_wall_	0.343	0.132	0.553	10.17	0.001
Endotracheal tube cuff inflated	-0.372	-0.737	-0.007	3.996	0.046

*T*_*airway*_ airway temperature; *CI* confidence interval; *T*_*tube_centre*_ temperature at the centre of the endotracheal tube; and *T*_*tube_wall*_ temperature at the wall surface of the endotracheal tube.

## Discussion

Our data from newborn piglets suggested that the inspiratory temperature of respiratory gases can be monitored using an ultra-rapid-response thermometer attached to the closed endotracheal tube suction system. With further validation, our airway thermometer might help optimise the humidity level of respiratory gases for mechanically ventilated patients and prevent complications associated with inappropriate humidification.

Appropriate heating and humidification of respiratory gases are important to prevent complications during mechanical ventilation^7^. Previous studies addressed the respiratory gas humidity using dummy respiratory circuits and artificial lungs^11–13^. However, there currently is no established method to monitor the temperature and humidity of respiratory gases at the distal end of the endotracheal tube. Haugk and colleagues proposed that periodic changes in the temperature measured on the outer surface of the endotracheal tube cuff might help predict the inspiratory gas temperature^14^. However, in our study, even T_tube_wall_, which was measured at the inner surface of the endotracheal tube, did not show significant temperature changes between inspiratory and expiratory phases, suggesting that the precise estimation of the inspiratory airway temperature would be difficult when the temperature is measured outside the endotracheal tube. Schiffmann and colleagues measured the respiratory gas humidity at the proximal end of the endotracheal tube using a specially designed hygrometer (sampling frequency, 20 Hz; time constant, unknown), which enabled the extraction of the inspiratory gas humidity from the expiratory gas humidity^15^. However, the inspiratory gas humidity at the proximal part of the endotracheal tube is clinically much less informative compared to that monitored at the distal end of the endotracheal tube^10^. In our study, we developed an airway thermometer by combining ultra-rapid-response thermocouples and a closed endotracheal tube suction system, which are commercially available and commonly used even in newborn infants^16–18^. By inserting a similar device into the endotracheal tube, temperature values which reflect the respiratory gas temperature at the mouth-corner level of the endotracheal tube, were monitored. Using the information provided by the manometer of the ventilator, we were able to identify the inspiratory and expiratory temperatures independently of each other. Although we used the simultaneous video recording to the graphic monitor of the ventilator, this information might be unnecessary because both T_airway_ and T_tube_centre_ showed distinct patterns between inspiratory and expiratory phases when the temperature values were plotted along time. However, considering that expiratory gas temperatures at the mouth-corner level theoretically mirror core body temperatures, lower body temperatures may be associated with smaller differences between inspiratory and expiratory gas temperatures, where the information from the manometer might be necessary to precisely identify the inspiratory phase. In addition, we initially expected that expiratory T_tube_centre_ reflects the core body temperature. However, expiratory T_tube_centre_ was higher than 38.5 °C, to which the target rectal temperature was servo-controlled. It is possible that the true core body temperature was much higher than the rectal temperature, because the radiant heat was mainly focused on the trunk of the piglet, and the rectal temperature is relatively lower than the oesophageal temperature in newborn species^19^.

We did not measure the airway temperature at the distal end of the endotracheal tube. This is based on an assumption that the inspiratory gas temperature theoretically goes down when travelling from the Y-piece to the mouth-corner level of the endotracheal tube, because the respiratory gases are cooled by the ambient air (26 °C in the current study), and this temperature level is much lower than the respiratory gas temperature at the Y-piece (set to 40 °C). Once the respiratory gases have passed the mouth-corner level, they are heated back to the body temperature (38.5 °C in newborn piglets and 37 °C in newborn infants)^20,21^. Therefore, the saturated vapour pressure calculated from the inspiratory gas temperature measured at the mouth-corner level (but not at the distal end of the endotracheal tube) would be suitable to estimate the humidity level delivered to the patient’s bronchus.

Given the relatively non-invasive nature of the device, our airway thermometer is likely to provide crucial information to prevent complications of invasive ventilation, which are related to the inappropriate humidification of respiratory gases. Latent but inappropriate humidification of critical levels have been reported in patients who are undergoing therapeutic hypothermia^22^. The optimal humidifier setting has not been established when the body temperature is controlled other than normothermic^23^. The temperature and humidity of inspiratory gases remain largely unknown when heat-moist exchangers are used instead of active humidifying systems^24^. Monitoring of the airway temperature at the mouth-corner level may help optimise the humidifier setting for such conditions as well as many others, where the risk of inappropriate humidification and subsequent problems are increased. However, caution is required because T_airway_ is unlikely to reflect humidity levels of inspiratory gases when respiratory gases have not been fully saturated at the outlet of the humidifying chamber^25^.

Our study simultaneously showed the possible limitations of our airway thermometer, which need to be addressed in future studies. First, the relationship between T_airway_ and T_tube_centre_ is likely to be affected by the presence of an endotracheal tube leakage. Indeed, deflation of the cuff was associated with higher T_airway_ presumably due to increased inspiratory gas flow, which is required to maintain the airway pressure. Second, because of technical reasons, the body temperature of the piglet was maintained at the normal body temperature for piglets, which is higher than that for human newborn infants. Third, due to ethical reasons, we were only able to study four piglets. Hence, we were able to assess the impact of limited clinical variables on the temperature reading of the airway thermometer. To compensate for the lack of a sufficient subject number, we tried to maximise the variation of the ventilator settings. We also repeated the temperature data acquisition with adequate time intervals, the influence of which was accounted for using the generalised estimating equation. Fourth, inspiratory gas temperatures were higher for T_airway_ than T_tube_centre_, suggesting the possibility that T_tube_centre_ was affected by the thermal exchange with the endotracheal tube. If this is correct, the true inspiratory gas temperature at the mouth corner level might better be reflected by T_airway_ rather than T_tube_centre_. Fifth, we were unable to incorporate T_Y-piece_ values within the analysis because of the technical problem, although we did not expect T_Y-piece_ to be a good surrogate for T_tube_centre_. Finally, all temperature data acquisitions were performed with spontaneous breathing attenuated by intravenous injections of a muscle relaxant. Therefore, the influence of spontaneous breathing needs to be assessed in future studies. Further preclinical and clinical studies are needed to validate the accuracy of the airway temperature estimation using the airway thermometer.

In newborn piglets, the inspiratory gas temperature travelling through the mouth-corner level of the endotracheal tube was successfully measured using an ultra-rapid-response thermometer attached to the closed endotracheal tube suction system. Using the measured temperature value, the condensation and humidity of the respiratory gases delivered to the bronchus of the newborn piglet can be calculated. With further validation, this relatively non-invasive airway thermometer may be useful for monitoring and optimising the humidity level of respiratory gases, and may contribute to the reduction of ventilation-associated complications. Further studies are required to validate the estimation of the inspiratory gas temperature in preclinical and clinical settings, where the influence of endotracheal tube leakage and spontaneous breathing needs to be assessed.

## Methods

### Study subjects and animal preparation

The protocol of this study was approved by the animal ethics committee of the Institute of Animal Experimentation, Kurume University School of Medicine, Fukuoka, Japan. The study was conducted in accordance with the principles of the Declaration of Helsinki. All the experiments were performed in compliance with the ARRIVE guidelines^26^. Four male newborn piglets (2 to 3 days old, 1.2 to 3.4 kg) were studied within 72 h of birth. The piglets were delivered from the farm in the morning of the experiment and sedated with an intramuscular injection of midazolam (0.2 mg/kg). Each piglet was placed on an open incubator and a peripheral venous catheter was inserted to give maintenance fluid (10% glucose, 80 ml/kg/day) and anaesthetics. The room was air-conditioned to maintain the ambient temperature of 26 °C. The electrocardiogram and percutaneous arterial oxygen saturation were monitored continuously, whereas non-invasive blood pressure and blood gases were assessed approximately every 30 min. General anaesthesia was then induced by 5% sevoflurane using a mask, and the piglet was intubated using a cuffed endotracheal tube with the internal diameter of 3 mm. The piglet was then placed in the left-lateral position and mechanically ventilated to maintain normal PCO_2_ using the pressure-control ventilation mode. Anaesthesia was maintained by 2–5% inhaled sevoflurane, a continuous intravenous infusion of dexmedetomidine (1 μg/kg/h) and intravenous pancuronium injections (0.7 mg/kg/dose, approximately every 30 min). The body temperature of the piglet was maintained at 38.5 ± 0.5 °C using a radiant heater, which was servo-controlled with rectal temperature feedback.

### Temperature monitoring

**Figure 3:**
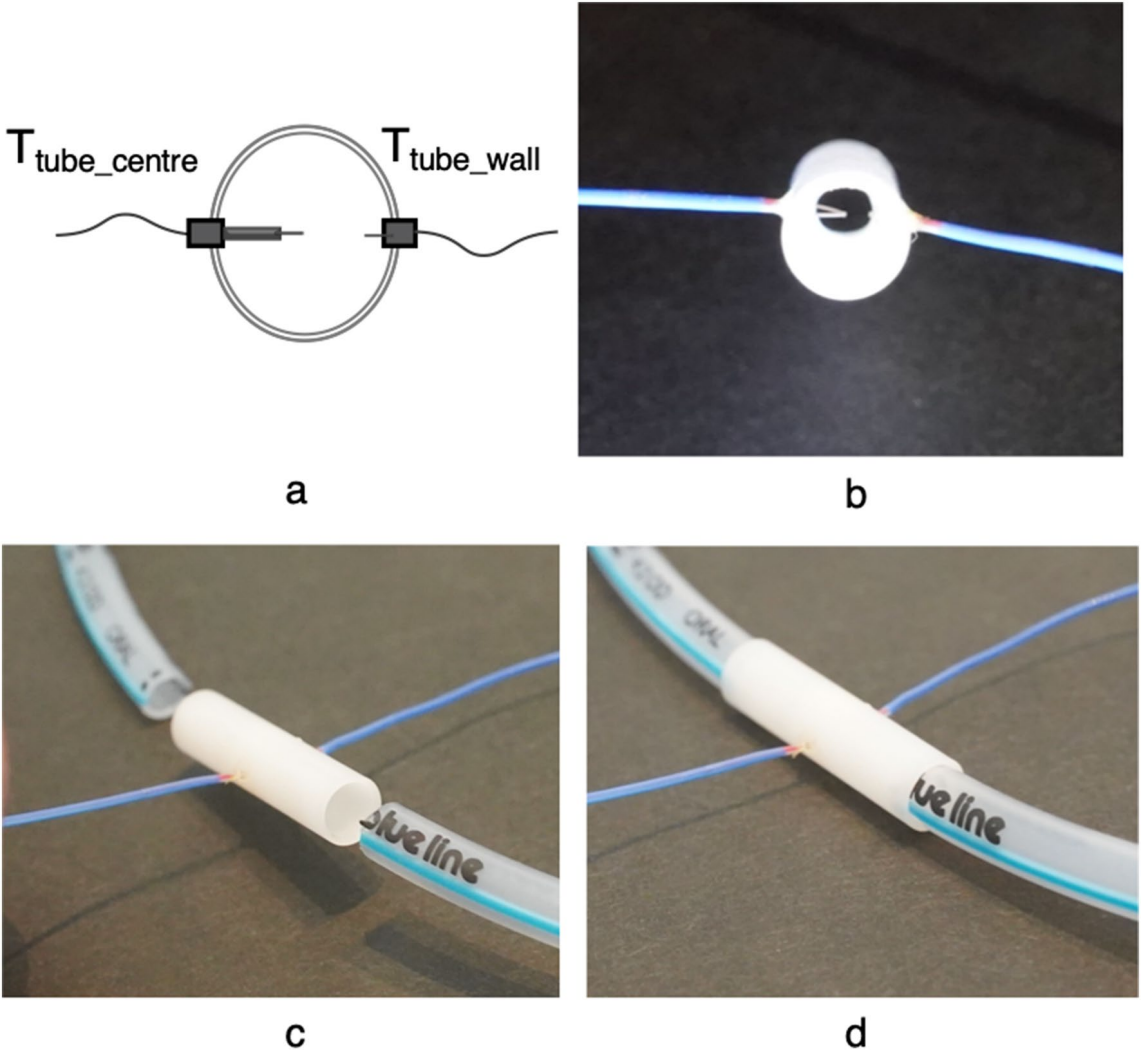
Diagram demonstrating the construction of an extension part of the endotracheal tube to monitor the respiratory gas temperature at the centre and the wall part of the tube. An extension part of the endotracheal tube was constructed by attaching two ultra-fast-response thermocouples [1.6 mm (T_tube_centre_) and 0.5 mm (T_tube_wall_)] from the endotracheal tube wall toward the centre of the endotracheal tube (**a**,**b**). This extension part was inserted between the distal and proximal parts of the endotracheal tube at the level of the mouth (**c**,**d**). *T*_*tube_centre*_ temperature at the centre of the endotracheal tube; and *T*_*tube_wall*_ temperature at the wall surface of the endotracheal tube.

**Figure 4:**
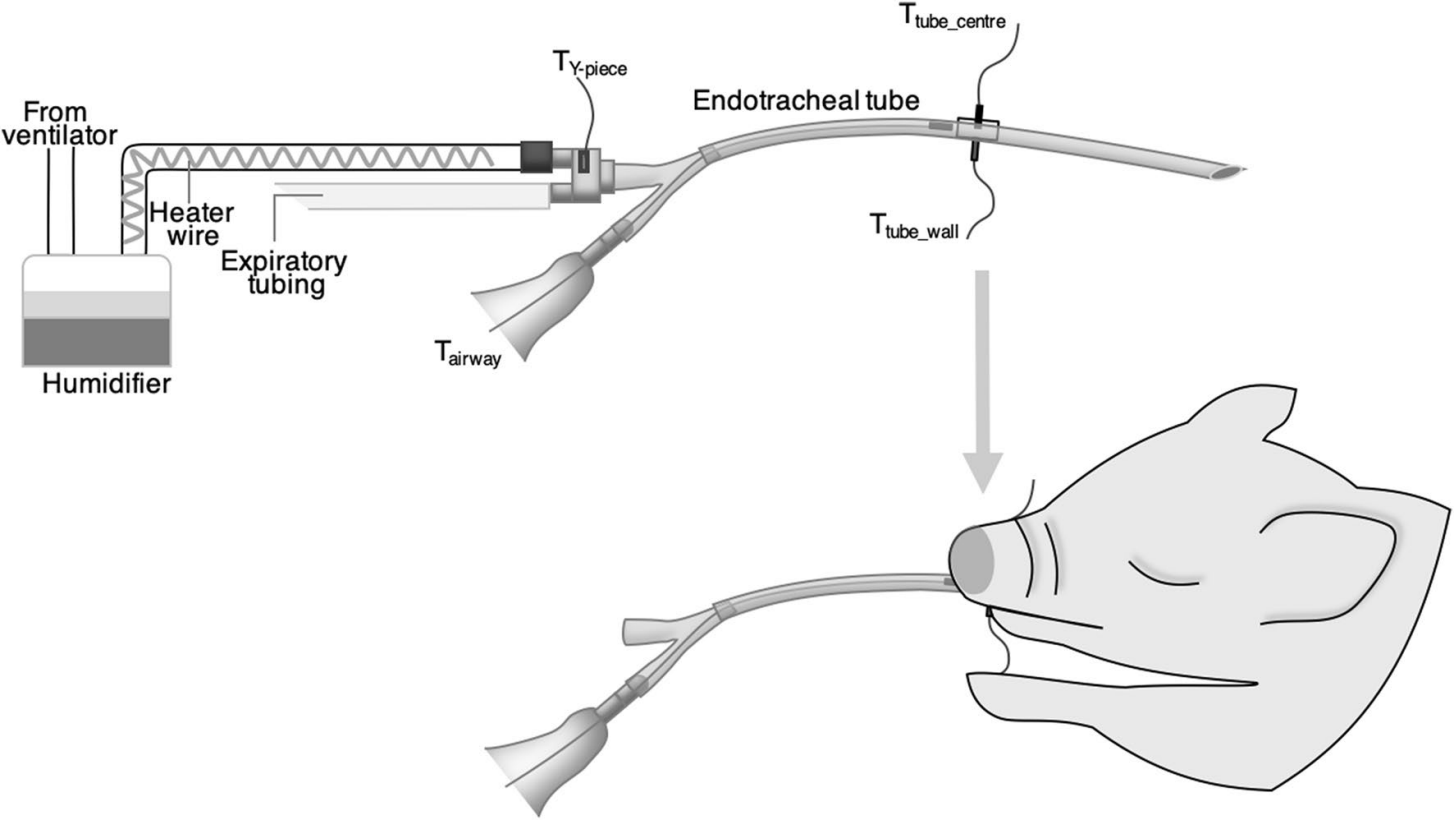
Diagrams depicting the configuration of the ventilator circuit and thermometers. An ultra-fast-response thermocouple was attached to the distal end of the closed suction system. Respiratory gas temperatures were measured using an airway thermometer (T_airway_) and other ultra-fast-response thermocouples attached to the Y-piece (T_Y-piece_) and the extension part of the endotracheal tube (see Fig. 3 for details).

A custom-made airway thermometer was developed using an ultra-fast-response thermocouple (time constant, 0.05 ms; FasTemp, Aivision, Tokyo, Japan) at the distal tip of the closed suction system (Ecocath, Medtronic plc., Dublin, Ireland). The respiratory gas temperature at the Y-piece of the ventilator circuit was monitored using a built-in thermometer of the humidifying system (MR 730, Fisher and Paykel, Auckland, New Zealand) and another FasTemp probe (T_Y-piece_). To assess the accuracy of the temperature measured using the airway thermometer (T_airway_), another custom-made system was developed by attaching two FasTemp probes to an 8 mm long tube with an internal diameter of 3.2 mm, 1.6 mm (T_tube_centre_) and 0.5 mm (T_tube_wall_) from the endotracheal tube wall to the centre of the endotracheal tube (Fig. 3); this unit was inserted into an endotracheal tube (internal diameter, 3 mm) once the animal was intubated and the endotracheal tube was cut at the level of the mouth (Fig. 4).

Prior to the experiment, each FasTemp probe was inspected so that temperature values are within ± 0.3 °C of the standard mercury thermometer using a water bath of 30 to 40 °C. Electronic signals from the thermocouples were transferred to a laptop computer through a data logger (C-Logger, Contec, Osaka, Japan) with a sampling rate of 200 Hz.

### Ventilators, circuits and humidifiers

We used a ventilator (Servo-300 ventilator, Siemens Elema, Solna, Sweden) with a humidifier and a disposable circuit (DAR Neonatal, Medtronic, Dublin, Ireland) which were configured based on the manufacturer’s recommendation (Fig. 4).

### Data collection

Temperature information was collected with the clocks of the data logging system, video camera and ventilator synchronised between each other prior to the experiment. Data acquisition was started before the insertion of the airway thermometer into the level of the mouth-corner, where the airway thermometer was withheld for at least 30 s, and was terminated after the removal of the airway thermometer (see Supplementary Fig. S2 online). Data acquisition was performed for a set of conditions with six different ventilator settings [combinations of PIPs (10, 15 and 20 cmH_2_O) and inspiratory times (0.5 and 1.0 s)] and with/without inflating the endotracheal tube cuff with 2.5 ml of air (cuff pressure was not measured since tracheal safety was not an issue in the current experiment); the pressure–volume curve of the ventilator graphic monitor was inspected to confirm that leakage at cuff/trachea interface was successfully eliminated by the cuff inflation.

The positive end-expiratory pressure (PEEP) and ventilation frequency were fixed to 5 cmH_2_O and 30 /min, respectively, whereas the humidifying chamber outlet and Y-piece temperatures were set to 37 °C and 40 °C, respectively. The data collection cycle (see Supplementary Fig. S1 online) was repeated up to three times with intervals of at least 30 min from the same piglet. After the completion of the study, piglets were euthanised by an overdose injection of intravenous phenobarbital.

### Data analysis

The time-trend data were assessed on a spreadsheet (Excel, Microsoft, DC, U.S.A.; MATLAB, Math Works, MA, U.S.A.) and classified into inspiratory and expiratory phases referring to the video images of the graphic monitor. Five breaths were chosen from the 30 s record per each ventilator setting to identify the highest expiratory temperature and lowest inspiratory temperature for T_airway_, T_tube_centre_ and T_tube_wall_, excluding 5 breaths immediately before and after ventilator setting changes. We also tried to extract inspiratory and expiratory T_Y-piece_, however, because of the intermittent high-frequency digital noise observed for the data from this channel, we decided not to consider T_Y-piece_ further.

The average of the five temperature values for each ventilator setting was used for the analysis. Independent variables to explain inspiratory T_airway_ were assessed using T_tube_centre_, T_tube_wall_, peak inspiratory pressure (10, 15 and 20 cmH_2_O), inspiratory time and the use of the balloon cuff (inflation and deflation). Univariate and multivariate generalised estimating equations were used to account for repeated sampling from the same piglet. This model was assessed with the Wald Chi-square test statistic with a level of significance set at p < 0.05.

## Supplementary Information

Supplementary Information.
