# Supplementary material for: Airway gas temperature within endotracheal tube can be monitored using rapid response thermometer
Source: Sci Rep. 2021 May 5;11:9537. doi: 10.1038/s41598-021-88787-3 (PMC8100119; doi:10.1038/s41598-021-88787-3)
Supplement: Supplementary file 1 — Supplementary Information. [file 41598_2021_88787_MOESM1_ESM.pdf]

Title:

Monitoring of airway gas temperatures within endotracheal tube using rapid-response thermometer

Short title: Monitoring of airway gas temperatures

Authors:

Shigeharu Nakane MD[1,2], Kennosuke Tsuda MD, PhD[1], Masahiro Kinoshita MD, PhD[3], Shin Kato MD, PhD[1], Sachiko Iwata MD, PhD[1], Yung-Chieh Lin MD[1,4], Mihoko Mizuno MD[2], Shinji Saitoh MD, PhD[1] and Osuke Iwata MD, PhD[1] *

Affiliations:

[1]Department of Paediatrics and Neonatology, Nagoya City University Graduate School of Medical Sciences, Nagoya, Japan.

[2]Department of Paediatrics, Daido Hospital, Nagoya, Japan.

[3]Department of Paediatrics and Child Health, Kurume University School of Medicine, Fukuoka, Japan.

[4]Department of Paediatrics, National Cheng Kung University Hospital, Tainan, Taiwan.

Supplementary Fig. S1:

Relationship between inspiratory T_{airway}, T_{tube_centre} and T_{tube_wall}.

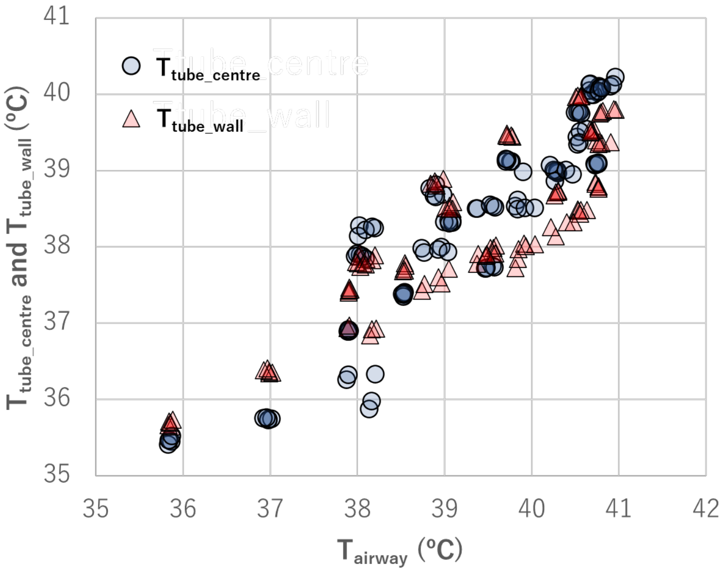

A representative scatter plot depicting the relationship between T_{airway} , T_{tube_centre} and T_{tube_wall} with a representative setting (peak inspiratory pressure, 10 cmH_2O; tracheal cuff, inflated; inspiratory time, 0.5 sec).

Abbreviations: T_{airway}, airway temperature; T_{tube_centre}, temperature at the centre of the endotracheal tube; and T_{tube_wall}, temperature at the wall surface of the endotracheal tube.

Supplementary Fig. S2:

Data acquisition cycles

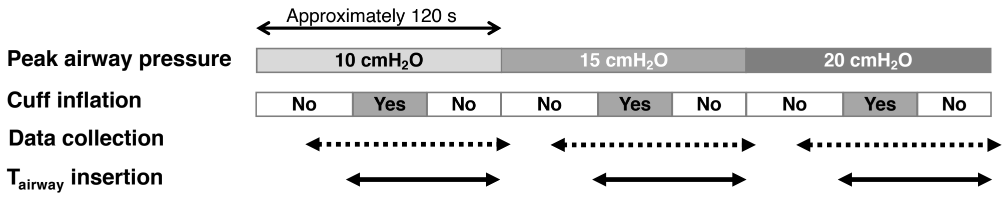

Data acquisition was performed for a set of different conditions which is combinations of peak inspiratory pressures (10, 15 and 20 cmH_2O) and with/without inflating the endotracheal tube cuff. This cycle was repeated at each inspiration times (0.5 and 1.0 sec).
